# Mangostanaxanthone IV Ameliorates Streptozotocin-Induced Neuro-Inflammation, Amyloid Deposition, and Tau Hyperphosphorylation via Modulating PI3K/Akt/GSK-3β Pathway

**DOI:** 10.3390/biology10121298

**Published:** 2021-12-08

**Authors:** Hossam M. Abdallah, Nesrine S. El Sayed, Alaa Sirwi, Sabrin R. M. Ibrahim, Gamal A. Mohamed, Nora O. Abdel Rasheed

**Affiliations:** 1 Department of Natural Products and Alternative Medicine, Faculty of Pharmacy, King Abdulaziz University, Jeddah 21589, Saudi Arabia; asirwi@kau.edu.sa (A.S.); gahussein@kau.edu.sa (G.A.M.); 2 Department of Pharmacognosy, Faculty of Pharmacy, Cairo University, Giza 11562, Egypt; 3 Department of Pharmacology and Toxicology, Faculty of Pharmacy, Cairo University, Giza 11562, Egypt; nesrine.salah@pharma.cu.edu.eg (N.S.E.S.); nora.osama@pharma.cu.edu.eg (N.O.A.R.); 4 Department of Chemistry, Preparatory Year Program, Batterjee Medical College, Jeddah 21442, Saudi Arabia; sabrin.ibrahim@bmc.edu.sa or; 5 Department of Pharmacognosy, Faculty of Pharmacy, Assiut University, Assiut 71526, Egypt; 6 Department of Pharmacognosy, Faculty of Pharmacy, Al-Azhar University, Assiut Branch, Assiut 71524, Egypt

**Keywords:** *Garcinia mangostana*, mangostanaxanthone IV, xanthones, neuro-inflammation, Alzheimer’s disease, PI3K/Akt/GSK-3β

## Abstract

**Simple Summary:**

Mangostanaxanthone IV (MX-IV) is a major constituent in *Garcinia mangostana*. It induced neuro-protective effects in the ICV-STZ mouse model through diminishing ICV-STZ-induced oxidative stress, neuro-inflammation, and apoptosis which was reflected by a significant reduction in malondialdehyde (MDA), hydrogen peroxide (H_2_O_2_), tumor necrosis factor-α (TNF-α), and interleukin-6 (IL-6) brain contents contrary to increased glutathione (GSH) content. It also decreased amyloid plaques’ number and phosphorylated *tau* expression via the PI3K/Akt/GSK-3β pathway.

**Abstract:**

Alzheimer’s disease (AD), a progressive neurodegenerative disorder, is characterized by amyloid deposition and neurofibrillary tangles formation owing to tau protein hyperphosphorylation. Intra-cerebroventricular (ICV) administration of streptozotocin (STZ) has been widely used as a model of sporadic AD as it mimics many neuro-pathological changes witnessed in this form of AD. In the present study, mangostanaxanthone IV (MX-IV)-induced neuro-protective effects in the ICV-STZ mouse model were investigated. STZ (3 mg/kg, ICV) was injected once, followed by either MX-IV (30 mg/kg/day, oral) or donepezil (2.5 mg/kg/day, oral) for 21 days. Treatment with MX-IV diminished ICV-STZ-induced oxidative stress, neuro-inflammation, and apoptosis which was reflected by a significant reduction in malondialdehyde (MDA), hydrogen peroxide (H_2_O_2_), tumor necrosis factor-α (TNF-α), and interleukin-6 (IL-6) brain contents contrary to increased glutathione (GSH) content. Moreover, nicotinamide adenine dinucleotide phosphate (NADPH) oxidase content and cleaved caspase-3 activity were reduced together with a marked decrement in amyloid plaques number and phosphorylated tau expression via PI3K/Akt/GSK-3β pathway modulation, leading to obvious enhancement in neuronal survival and cognition. Therefore, MX-IV is deemed as a prosperous nominee for AD management with obvious neuro-protective effects that were comparable to the standard drug donepezil.

## 1. Introduction

Alzheimer’s disease (AD), a neurodegenerative disease with increasing prevalence with age, is portrayed by progressive cognitive dysfunction [1]. The exact etiology of AD remains unknown despite the advances in our knowledge regarding the disease pathology. Oxidative stress, neuro-inflammation, insulin resistance, and synaptic failure along with amyloid-beta (Aβ) deposition and tau hyperphosphorylation are involved in the pathogenesis of sporadic AD (SAD), the most prevalent form of AD [2]. Intra-cerebroventricular (ICV) injection of streptozotocin (STZ), in a sub diabetogenic dose, in rodents was reported to induce significant behavioral and pathological changes that resemble humans’ SAD [3]. Studies have shown that ICV-STZ injection was accompanied by oxidative stress, glial activation, spatial memory impairments in addition to tau hyperphosphorylation, and Aβ deposition [4,5]. Oxidative stress is reported to play a crucial role in AD pathology and progression as it contributes to Aβ aggregation and tau hyperphosphorylation which provokes further redox imbalance [6]. Nicotinamide adenine dinucleotide phosphate (NADPH) oxidase is an enzyme complex responsible for the generation of reactive oxygen species (ROS) by the microglia. Thus, its upregulation is associated with AD-induced neurotoxicity via the direct toxic effect of ROS and also as ROS tend to potentiate the generation of several pro-inflammatory and neuro-toxic cytokines, such as tumor necrosis factor-alpha (TNF-α) [7]. Interleukin-6 (IL-6) and TNF-α were reported to increase the Aβ burden and promote tau abnormal phosphorylation. Thus, oxidative stress and neuro-inflammation are deemed as key players in Aβ deposition and tau hyperphosphorylation which are the main hallmarks associated with AD [8]. The phosphoinositide 3-kinase (PI3K)/protein kinase B (Akt) signaling pathway is one of the most vital pathways for neuronal survival and it is also involved in neuro-inflammation [9].

Activation of PI3K promotes Akt phosphorylation and activation, then p-Akt phosphorylates and inhibits glycogen synthase kinase-3β (GSK-3β), which is a downstream kinase of PI3K/Akt. GSK-3β is implicated in microglial activation and the generation of various pro-inflammatory cytokines [10]. Moreover, it induces the activation of many protein kinases involved in apoptosis. Additionally, GSK-3β is known to contribute to increased tau protein phosphorylation which eventually leads to neurofibrillary tangles formation [11]. Consequently, AD is a complex neurodegenerative disorder distinguished by Aβ aggregation and tau hyper-phosphorylation along with oxidative stress, neuro-inflammation, and neurodegeneration with obvious cognitive decline [12].

Recently, complementary and herbal medicine have gained popularity and interest. In this regard, many plant-based compounds showed promising anti-Alzheimer activity [5]. It was reported that the consumption of flavonoids and flavonoids-rich food could enhance cognitive abilities via their protective effects against free radical-induced neuronal damage [13]. Additionally, green tea, cocoa, blue berry, and other flavonoid rich food contributed to cognitive enhancement in AD animal models [14,15].

In this regard, xanthones such as α-mangostin showed multifunctional activities for the treatment of Alzheimer’s disease [16]. *Garcinia mangostana* L. (mangosteen, the queen of fruits) is known for its eatable fruit that was traditionally used to treat ulcer, diarrhea, fever, hypertension, obesity, and diabetes [17]. The rind of the fruit is rich in xanthones that have multiple biological activities [18,19,20,21,22,23]. MX-IV is a new xanthone isolated for the first time from *G. mangostana* that was able to inhibit the formation of advanced glycation end products in vitro and induced apoptosis in colorectal adenorectal carcinoma cells (HCT-116) [17,24]. Due to the lack of data on biological activities of this new compound and based on the various activities of xanthones [25] and the similarity of MX-IV to α- mangostin, the present study was conducted to investigate the possible neuro-protective effects of MX-IV isolated from *G. mangostana* in ICV-STZ-induced AD in mice.

## 2. Materials and Methods

### 2.1. Isolation and Purification of MX-IV

MX-IV was isolated from the rind of *G. mangostana* fruit following the same procedures described before [24]. Briefly, dried powder pericarp of *G. mangostana* was extracted with methanol and the dried extract was suspended in water and fractionated with chloroform. Chloroform fraction was fractionated on silica gel column using hexane:EtOAc (90:10) to give MX-IV.

### 2.2. Animals

Adult male Swiss albino mice (25–30 g, 3–4 months) were used. Animals were obtained from the animal house facility of the Faculty of Pharmacy Cairo University. Mice were accommodated at 10 per cage in polycarbonate cages with free access to food and water. The study was approved by the Institutional animal care and use committee (CU-IACUC) (approval number: CU III F 27 20) with the recommendations of the National Institutes of Health Guide for Care and Use of Laboratory Animals (2011) being fulfilled.

### 2.3. General Experimental Procedures

Induction of SAD was carried out as previously described by Rasheed et al. in 2018 [2]. Streptozotocin (STZ) and donepezil (Sigma–Aldrich, St. Louis, MO, USA) were dissolved in 0.9% saline [26,27]. Mice were randomly distributed into five groups each containing 10 mice. Group I received 0.9% saline ICV as well as distilled water with 5% carboxymethylcellulose oral and deemed as the normal group, while group II received MX-IV (30 mg/kg/day, oral) [28]. Group III was injected with STZ 3 mg/kg, ICV) once [2]. Groups IV and V received STZ (3 mg/kg, ICV) once, then after 5 h, group IV was injected with MX-IV (30 mg/kg/day, oral) while group V received donepezil (2.5 mg/kg/day, oral) [3]. Both MX-IV and donepezil were injected daily for 21 days. Morris water maze test was carried out one day following the end of the injection schedule.

### 2.4. Morris Water Maze (MWM) Test

The MWM test traces the animals’ learning and visuospatial cognitive functions [29]. The procedure was performed on five successive days, as formerly described by [2]. The mean escape latency (MEL) and the time consumed by each mouse in the target quadrant on the test day were recorded as stated previously [30].

### 2.5. Tissue Sampling

After the MWM test was performed, mice were euthanized by cervical dislocation and decapitation. The brains were rapidly excised and washed with ice-cold saline. The brain tissues were then weighed and homogenized in saline to prepare 10% homogenates. After centrifugation, the supernatants were used for measurement of nicotinamide adenine dinucleotide phosphate (NADPH) oxidase activity in addition to brain malondialdehyde (MDA), reduced glutathione (GSH), hydrogen peroxide (H_2_O_2_), tumor necrosis factor-alpha (TNF-α), and interleukin-6 (IL-6) contents.

### 2.6. Estimation of Biochemical Parameters

TNF-α was assessed by means of a mouse ELISA kit (Cusabio, Wuhan, China). Additionally, GSH, MDA, H_2_O_2,_ IL-6, and NADPH oxidase brain contents were quantified using the corresponding mouse ELISA kits (My Bio Source, San Diego, CA, USA).

### 2.7. Western Blot Analysis 

Estimation of brain p-tau, p-PI3K, p-Akt, p-GSK-3β, and cleaved caspase-3 expression was carried out by Western blot analysis. Brain tissue (100 mg) was homogenized with RIPA buffer, and the lysates were centrifuged. Upon proteins denaturation, samples were loaded onto individual lanes on sodium dodecyl sulfate-polyacrylamide gel electrophoresis (SDS-PAGE) with β-actin protein being also loaded as a marker protein. Gels were transferred to nitrocellulose membranes subsequent to electrophoresis, then the membranes were incubated with 1:1000 dilutions of the following primary antibodies: anti-p-PI3K, anti-p-Akt, anti-p-GSK-3β, anti-cleaved caspase 3 (Cell Signaling Technology, Danvers, MA, USA), and anti-p-tau (Termo Fisher Scientific, Waltham, MA, USA). Subsequently, the membranes were probed with the secondary peroxidase-labelled antibodies (Dianova, Hamburg, Germany). Finally, the protein bands were developed, and their intensities were then quantified by densitometric analysis, with the results being presented as arbitrary units relative to the intensity of the corresponding β-actin bands.

### 2.8. Histopathological Examination

Neutral buffer formalin was used for the fixation of the brains of 3 mice for at least 48 h. Afterwards, they were washed, dehydrated in alcohol, and embedded in paraffin blocks. Tissue sections, 4 μm thickness, were stained with hematoxylin and eosin stain (H&E) for initial histopathological examination. Moreover, Congo red stain was used to reveal amyloid plaques. Ten random microscopic fields (20×) were used in the quantification of amyloid plaques, according to the method of Snowdon [31]. To evaluate the neuronal loss, the surviving neurons in the cerebral cortex and the hippocampal regions, cornu ammonis (CA3&4), and dentate gyrus (DG) were quantified according to the method of West et al. [32]. The neuronal survival rate was assessed as the percentage of intact neurons. Nissl-stained sections of the cerebral cortex and the hippocampus (CA3, CA4, and DG) were used for the assessment of neurodegeneration. Damaged neurons were indicated as those with shrunken cell bodies and condensed cytoplasm [33].

### 2.9. Statistical Analysis

The mean escape latency in MWM trials was assessed by repeated-measures analysis of variance (ANOVA) while the rest of the study results were analyzed using one-way ANOVA followed by Tukey’s multiple comparison test. All data were presented as mean ± SEM. Statistical analysis was performed using the GraphPad Prism© software (version 6.01; Graph Pad Software, San Diego, CA, USA) with the level of significance fixed at *p* < 0.05.

## 3. Results

### 3.1. Identification of Isolated Compound

**Figure 1:**
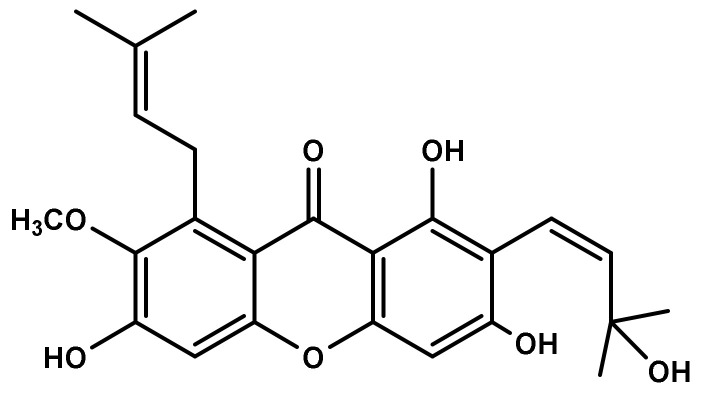
Chemical structure of Mangostanaxanthone IV (MX-IV).

Identification of MX-IV (Figure 1) was accomplished using different spectroscopic data including one- and two-dimension NMR, as previously described [24]. All detailed methods of identification are presented in the Supplementary Materials.

### 3.2. Effect of MX-IV on ICV-STZ-Injected Mice Behavior in Morris Water Maze Task

**Figure 2:**
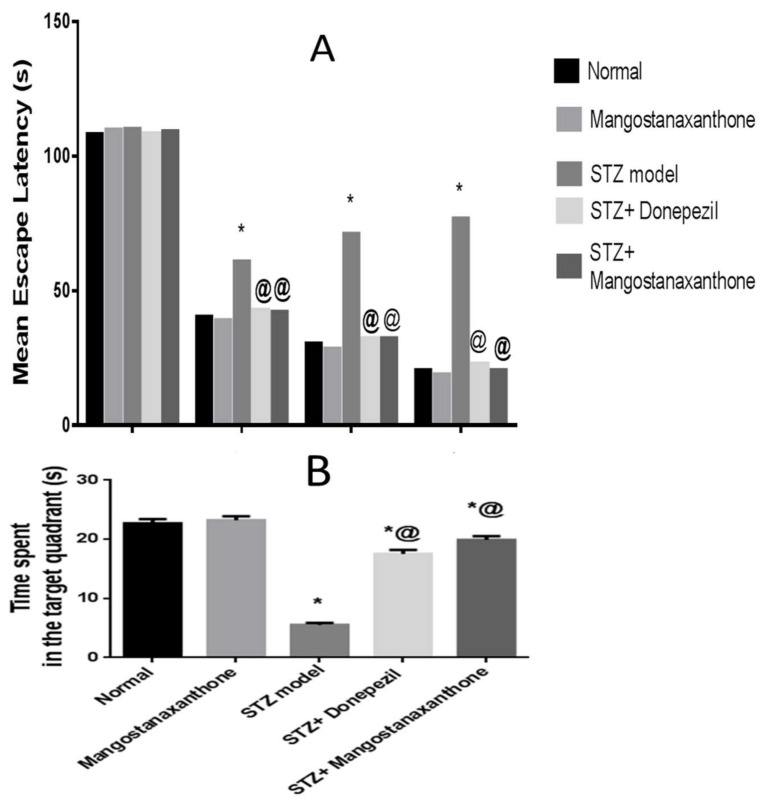
Effect of MX-IV on ICV-STZ-injected mice behavior in Morris water maze task. (**A**) The mean escape latency (MEL) and (**B**) the time spent in the target quadrant in Morris water maze task in ICV-STZ- injected mice. Values are expressed as mean ± SEM; *n* = 10. * *p* < 0.05 vs. normal group, @ *p* < 0.05 vs. ICV-STZ group.

On the first training day, there was no substantial variation in the MEL among all groups. Mice in all groups took less time to reach the platform starting from the second training day, with the ICV-STZ group showing the uppermost MEL. There was no marked difference in the MEL among normal mice whether treated with MX-IV or not. The MEL of the ICV-STZ group revealed an obvious increment. ICV-STZ injected mice which received either MX-IV or donepezil showed significant amelioration in the MEL on days 2, 3, and 4 with no significant difference between the treated groups. On the test day, animals treated with MX-IV showed no major difference in the time consumed in the target quadrant, as compared with normal mice. ICV-STZ group revealed an obvious decrease in the time spent in the target quadrant, whereas it was markedly increased in treated ICV-STZ groups. Thus, MX-IV administration to the ICV-STZ group revealed significant enhancement in spatial memory comparable to the standard reference donepezil (Figure 2).

### 3.3. Effect of MX-IV on Prominent Oxidative Stress Owing to ICV-STZ Injection

**Figure 3:**
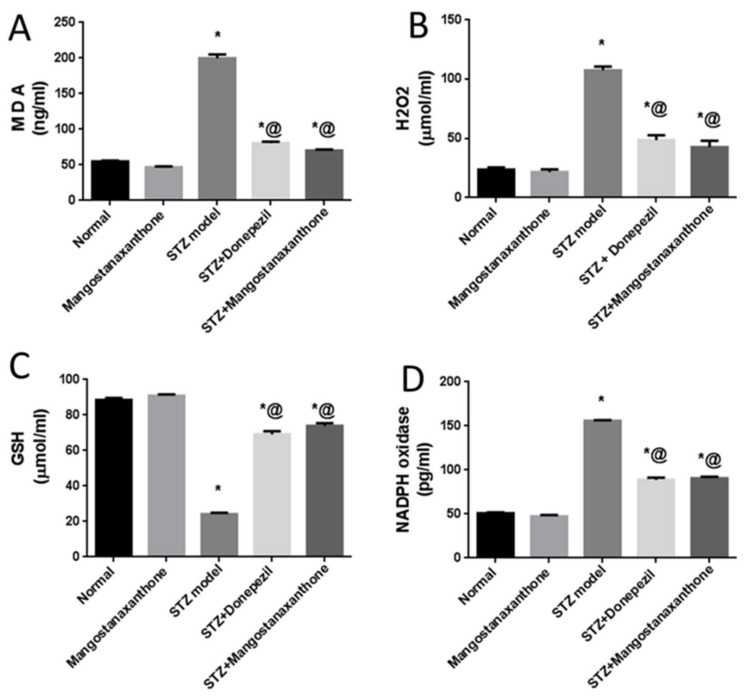
Effect of MX-IV on prominent oxidative stress owing to ICV-STZ injection. (**A**) MDA; (**B**) H_2_O_2_; (**C**) GSH; (**D**) NADPH oxidase. Values are expressed as mean ± SEM; *n* = 7. * *p* < 0.05 vs. normal group, @ *p* < 0.05 vs. ICV-STZ group.

Administration of MX-IV in normal mice showed no significant difference in reduced glutathione (GSH), malondialdehyde (MDA), and hydrogen peroxide (H_2_O_2_) brain contents as well as nicotinamide adenine dinucleotide phosphate (NADPH) oxidase activity, as compared with the normal control group. Injection of ICV-STZ contributed to increased oxidative stress reflected as reduced GSH content contrary to elevated MDA and H_2_O_2_ contents along with NADPH oxidase enhanced activity. Treatment with MX-IV or donepezil diminished ICV-STZ-induced oxidative stress with increased GSH content in contrast to decreased MDA and H_2_O_2_ contents and reduced NADPH oxidase activity. Consequently, MX-IV retarded ICV-STZ-induced oxidative damage as the reference drug donepezil (Figure 3).

### 3.4. Effect of MX-IV on Neuro-Inflammation Linked to ICV-STZ-Injection

**Figure 4:**
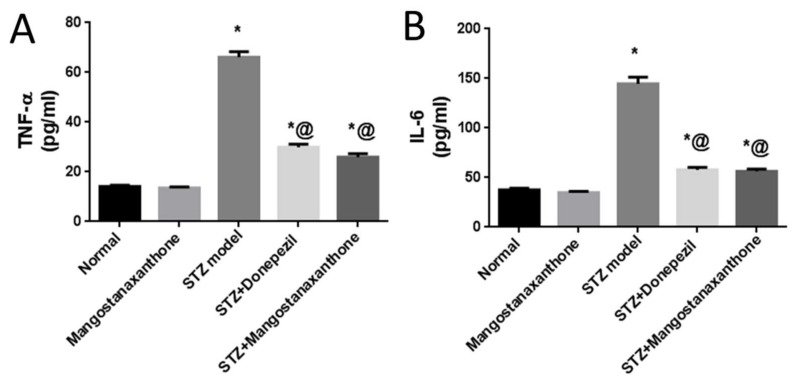
Effect of MX-IV on neuro-inflammation linked to ICV-STZ-injection. (**A**) TNF-α; (**B**) IL-6. Values are expressed as mean ± SEM; *n* = 7. * *p* < 0.05 vs. normal group, @ *p* < 0.05 vs. ICV-STZ group.

Administration of MX-IV in normal mice showed no significant difference in the brain contents of tumor necrosis factor-alpha (TNF-α) and interleukin-6 (IL-6), as compared with the normal control group. ICV-STZ injection induced an increase in the formerly mentioned pro-inflammatory cytokines. Treatment with either MX-IV or donepezil ameliorated neuro-inflammation associated with ICV-STZ administration with decreased TNF-α and IL-6 contents. Thus, MX-IV overcame ICV-STZ-induced neuro-inflammation like the reference drug donepezil (Figure 4).

### 3.5. Effect of MX-IV on ICV-STZ-Induced Disruption in p-PI3K, p-Akt, p-GSK-3β, p-tau Protein, and Cleaved Caspase-3 Expression

**Figure 5:**
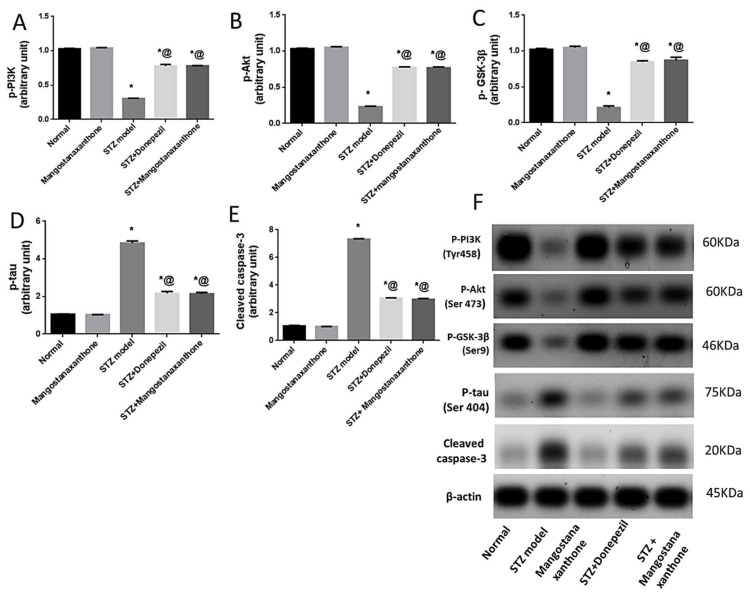
Effect of MX-IV on ICV-STZ-induced disruption in (**A**) p-PI3K; (**B**) p-Akt; (**C**) p-GSK-3β; (**D**) p-tau protein, and (**E**) cleaved caspase-3 expression (**F**) Western blot analysis of p-PI3K, p-Akt, p-GSK-3β, p-tau protein, and cleaved caspase-3 levels. Values are expressed as mean ± SEM; *n* = 7. * *p* < 0.05 vs. normal group, @ *p* < 0.05 vs. ICV-STZ group. Please refer to the full western blot in the Supplementary Materials.

Administration of MX-IV in normal mice showed no significant difference in p-PI3K, p-Akt, p-GSK-3β, p-tau protein, and cleaved caspase-3 protein expression, as compared with the normal control group. ICV-STZ injection promoted the expression of p-tau protein and cleaved caspase-3, whereas p-PI3K, p-Akt, and p-GSK-3β protein expression was inhibited. Treatment with MX-IV or donepezil reversed ICV-STZ-induced deleterious effects with upregulation of p-PI3K, p-Akt, and p-GSK-3β contrary to the decreased expression of cleaved caspase-3 and p-tau proteins. Thus, MX-IV inhibited ICV-STZ-induced aberrant PI3K/Akt/GSK-3β signaling, abnormal tau phosphorylation, and apoptosis almost equally as the reference drug donepezil (Figure 5).

### 3.6. Effect of MX-IV Administration on ICV-STZ-Injected Mice Brain Histopathological Examination

**Figure 6:**
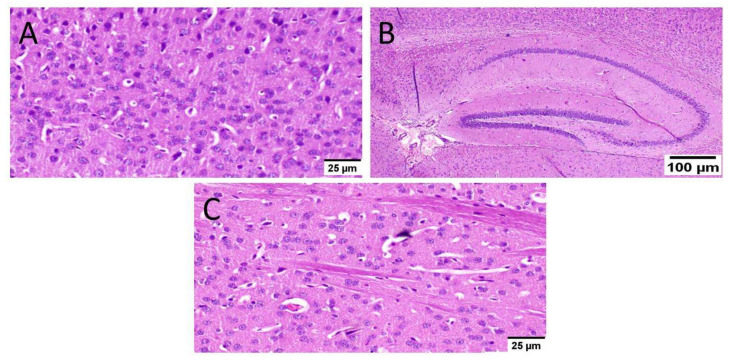
Histopathological examination of normal mice brain (*n* = 3) (H&E × 400). Normal mice (group I) showed normal histological structure of different neurons in the (**A**) cerebral cortex, (**B**) the hippocampus, and(**C**) the striatum.

Normal mice (group I) showed normal histological structure of different neurons in the cerebral cortex, the hippocampus, and the striatum (Figure 6).

**Figure 7:**
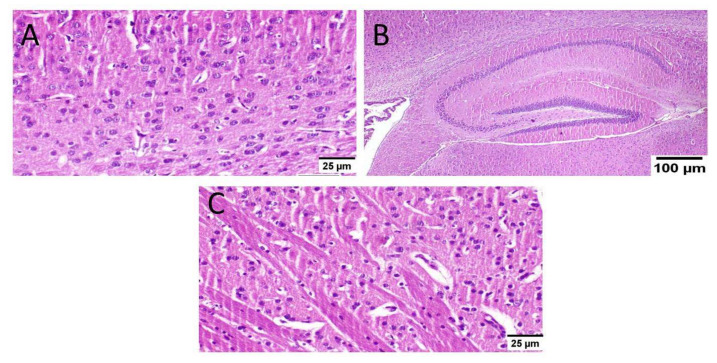
Histopathological examination of the brain of normal mice which received MX-IV (*n* = 3) (H&E × 400). Normal mice receiving MX-IV (group II) showed normal histological structure of different neurons in the (**A**) cerebral cortex, (**B**) the hippocampus, and(**C**) the striatum.

As well, mice that received MX-IV (group II) revealed normal histology in all anatomical sites of the brain tissue including the cerebral cortex, the hippocampus, and the striatum (Figure 7).

**Figure 8:**
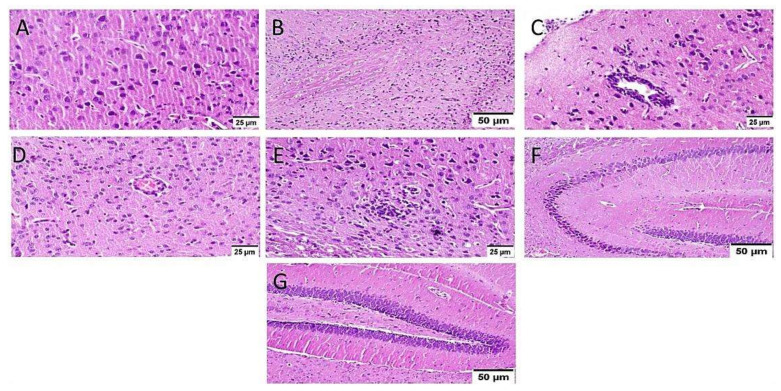
Histopathological alteration induced by ICV-STZ injection (*n* = 3) (H&E × 400). Mice injected with ICV-STZ (group III) showed (**A**) numerous scattered dark degenerated neurons of the cerebral cortex (**B**) and the striatum. The cerebral cortical blood vessels also exhibited (**C**) severe vasculitis associated with neuronal degeneration and neuronophagia (**D**) congestion with diffuse gliosis and (**E**) focal lymphocytic infiltration with degenerated neurons. The hippocampus showed numerous dark degenerated neurons of (**F**) cornu ammonis (CA) 3&4 as well as (**G**) dentate gyrus (DG) regions.

ICV-STZ injection (group III) resulted in diffuse gliosis with numerous scattered dark degenerated neurons in the cerebral cortex and the striatum. The cerebral cortical blood vessels also exhibited severe vasculitis and congestion associated with neuronal degeneration and neuronophagia with multifocal aggregations of lymphocytic infiltration. The hippocampus showed numerous dark degenerated neurons of cornu ammonis (CA) 3&4 as well as dentate gyrus (DG) regions (Figure 8).

**Figure 9:**
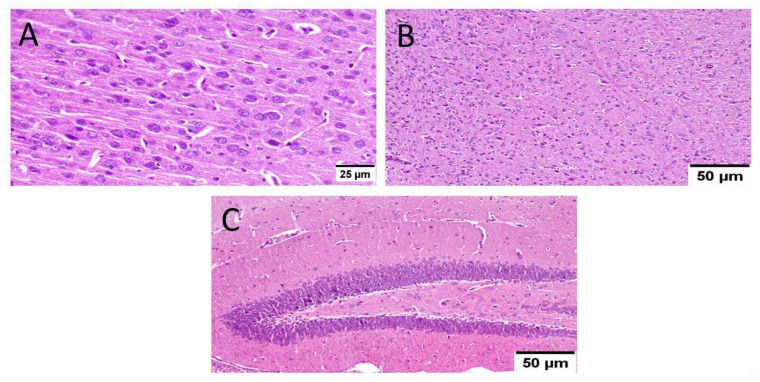
Effect of MX-IV on ICV-STZ injected mice brain histopathological examination (group IV) (*n* = 3) (H&E × 400). (**A**) The cerebral cortex showed apparently normal neurons. (**B**) The striatum revealed diffuse gliosis in some examined sections. (**C**) The hippocampus showed apparently normal neurons in all examined regions, except for DG regions that showed few scattered dark degenerated neurons.

Administration of MX-IV reduced the adverse effect of STZ injection in group IV. The cerebral cortex showed apparently normal neurons. The striatum showed diffuse gliosis in some examined sections; meanwhile, other sections exhibited apparently normal histological structure. The hippocampus showed apparently normal neurons in all examined regions, except for DG regions that revealed few scattered dark degenerated neurons (Figure 9).

**Figure 10:**
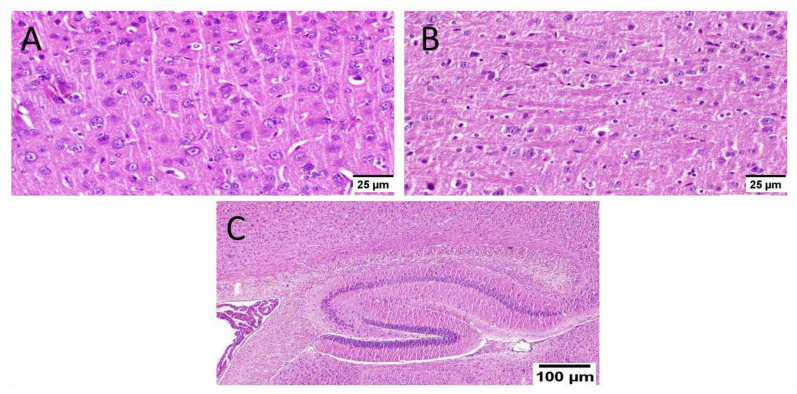
Effect of donepezil on ICV-STZ injected mice brain histopathological examination (*n* = 3) (H&E × 400). Animals treated with donepezil in group V showed (**A**) apparently normal neurons of the cerebral cortex. (**B**) The striatum showed diffuse gliosis in some instances. (**C**) The hippocampus showed apparently normal neurons in all examined sites.

Animals treated with donepezil in group V revealed apparently normal neurons of the cerebral cortex. Meanwhile, examination of striatum showed diffuse gliosis in some instances. The hippocampus showed apparently normal neurons in all examined sites (Figure 10).

### 3.7. Effect of Administration of MX-IV on Neuronal Survival Rate

**Figure 11:**
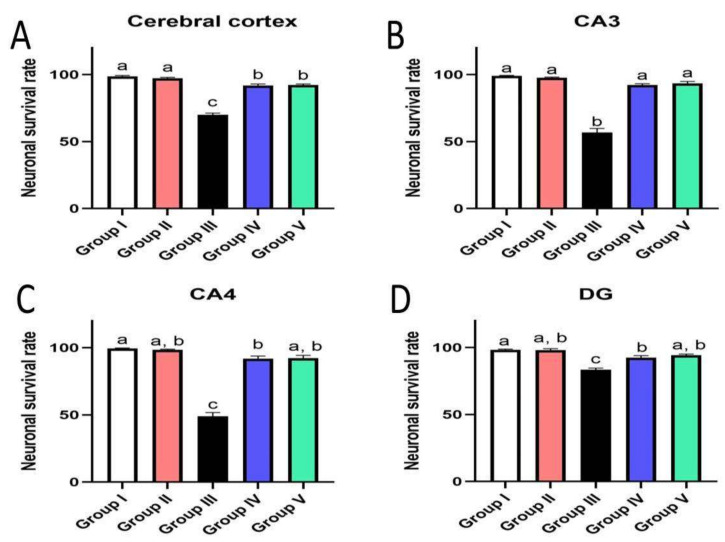
Effect of administration of MX-IV on neuronal survival rate (*n* = 3). Neuronal survival rate of different experimental groups was examined in (**A**) the cerebral cortex, (**B**) CA3, (**C**) CA4, and (**D**) DG regions. Values are expressed as mean ± SEM; *n* = 3. Different letters (a–c) above the error bar indicate statistically significant differences at *p* < 0.05.

**Figure 12:**
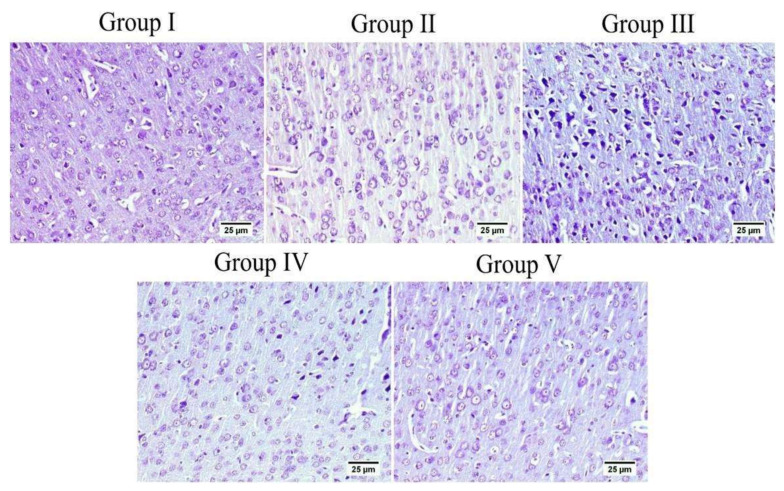
The cerebral cortex of different groups stained with Nissl stain. Normal group (group I) and mice injected with MX-IV (group II) showed normal healthy neurons. ICV-STZ group (group III) revealed numerous affected neurons. ICV-STZ-injected mice which received MX-IV (group IV) showed a fewer number of degenerated neurons. Donepezil-treated ICV-STZ-injected mice (group V) showed apparently normal cortex.

**Figure 13:**
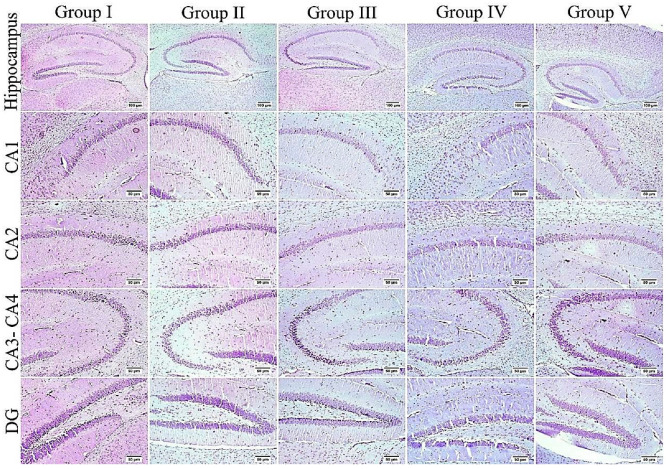
The hippocampus of different groups stained with Nissl stain. An elevated number of dark and shrunken neurons with pyknotic nuclei was detected in the ICV-STZ group (group III) especially in CA3, CA4, and DG regions; otherwise, the remaining experimental groups mostly exhibited normal intact neurons in all hippocampal regions.

The survival rate of neurons was examined in the cerebral cortex and the hippocampus. The results showed an obvious decrease in the cell survival rate of mice injected with ICV-STZ (group III) in all examined brain sites compared to other experimental groups. Administration of either the xanthone or donepezil increased the neuronal survival, as compared with the ICV-STZ group. A marked reduction was seen in the treated groups in comparison with normal mice and mice injected with MX-IV (group I and II, respectively) in the cerebral cortex. Meanwhile, no significant difference was recorded between treated groups and group I or group II in the CA3 region of the hippocampus. Concerning CA4 and DG hippocampal regions, no significant difference was found in groups II and the treated groups; however, a mild significant decrease was found in ICV-STZ injected animals that received MX-IV (group IV), as compared with the normal control group (group I). The morphology of the affected neurons appeared dark, shrunken, and degenerated in the cerebral cortices and the hippocampus of different groups (Figure 11, Figure 12 and Figure 13).

### 3.8. Effect of Administration of MX-IV on Amyloid Plaques Number

**Figure 14:**
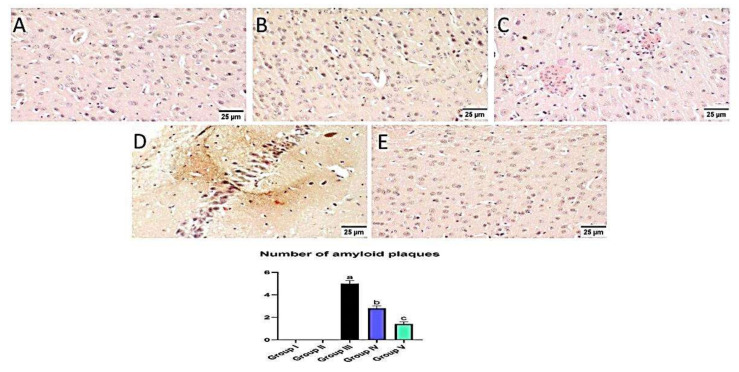
Effect of administration of MX-IV on amyloid plaques number. Congo red stain was used for detection of amyloid plaques. Normal mice showed absence of amyloid deposition in the brain tissue whether the (**A**) control group or (**B**) those injected with MX- (groups I and II). (**C**) Large and multifocal depositions of amyloid plaques surrounded by focal inflammatory reaction were detected in group III which received ICV-STZ injection. (**D**) Administration of MX-IV resulted in a marked reduction in the number and size of amyloid plaques in the brain tissue. (**E**) Group V, which was injected with donepezil, showed few to absence of amyloid plaques in most examined brain tissue sections. Values expressed as mean ± SEM; *n* = 3. Different letters (a–c) above the error bar indicate statistically significant differences at *p* < 0.05.

Normal animals showed no amyloid deposition in the brain tissue whether they received MX-IV or not (group II and I, respectively). However, large and multifocal areas of amyloid deposition associated with inflammatory tissue response were detected in group III which received ICV-STZ injection. Administration of MX-IV resulted in a noticeable decline in the number and size of amyloid plaques in the brain tissue. Group V, which was injected with donepezil, showed few to absence of amyloid plaques in most of the investigated sections (Figure 14).

## 4. Discussion

The present investigation was performed to inspect MX-IV possible neuro-protective effects against ICV-STZ-induced AD in mice. ICV-STZ injection is an acknowledged model for mimicking sporadic Alzheimer’s disease (SAD) in humans [2]. In the present study, mice that received ICV-STZ displayed depreciated cognitive functions reflected by the substantial increment in the mean escape latency (MEL) contrary to spending a short period of time in the target quadrant on Morris water maze (MWM) trials and test day, respectively. Similarly, previous studies reported suboptimal performance in MWM task associated with ICV-STZ injection [2,34]. Administration of MX-IV induced an apparent enhancement in cognitive abilities of ICV-STZ-injected mice, reflected as prominent MEL reduction contrary to marked increment in the time spent in the target quadrant on MWM trials and the test day, respectively. This marked cognitive improvement associated with MX-IV treatment appeared to be comparable to the standard reference drug donepezil. The interplay between oxidative stress, inflammation, and amyloid-beta (Aβ) plaques deposition was reported to contribute to AD pathology and progression [35]. ICV-STZ injection was known to induce oxidative stress, lipid peroxidation, neuro-inflammation, and Aβ deposition [2,35]. Likewise, in the present study, ICV-STZ-injected animals revealed a decreased level of the antioxidant reduced glutathione (GSH) while the lipid peroxidation marker, malondialdehyde (MDA), was significantly increased, as compared with normal mice. Hydrogen peroxide (H_2_O_2_) level was also increased in mice receiving ICV-STZ injection which reflects oxidative damage, as it can be converted into highly reactive free radicals, namely hypochlorous acid and hydroxyl radical that are involved in various pathological conditions [36]. Nicotinamide adenine dinucleotide phosphate (NADPH) oxidase, a chief source of reactive oxygen species, is involved in ICV-STZ -induced AD-like state, as its deletion attenuated STZ-induced neuro-inflammation, tau phosphorylation, and Aβ accumulation [37].

In the current study, the ICV-STZ group also exhibited prominent increase in NADPH oxidase activity in comparison with the normal mice. Administration of MX-IV in normal animals showed no substantial difference in the levels of the formerly mentioned parameters, as compared with normal control mice. Treatment with MX-IV or donepezil combated ICV-STZ induced oxidative stress reflected by reduced MDA and H_2_O_2_ levels along with NADPH oxidase reduced activity contrary to GSH increased level with MX-IV being nearly equally effective as donepezil. Oxidative stress is implicated in the generation of numerous pro-inflammatory cytokines such as, tumor necrosis factor-alpha (TNF-α) and interleukin-6 (IL-6) which contribute to further redox imbalance leading to a vicious cycle [38]. Neuro-inflammation is implicated in the induction of the amyloidogenic pathway of amyloid precursor protein processing in addition to increased tau protein phosphorylation [39]. It was reported that modulation of the phosphoinositide 3-kinase (PI3K)/protein kinase B(Akt)/glycogen synthase kinase-3β(GSK-3β) pathway retards ICV-STZ-induced neuro-inflammation, tau hyperphosphorylation, and cognitive decline [10]. GSK-3β is rendered inactive via its phosphorylation by activated phosphorylated Akt, thus inhibiting its hazardous effects such as stimulation of tau hyperphosphorylation and Aβ deposition, enhancement of microglial pro-inflammatory cytokines release, and induction of apoptosis [40]. Similarly, in the present study, the ICV-STZ group showed a significant elevation in the levels of pro-inflammatory cytokines TNF-α as well as IL-6 in addition to increased p-tau protein and cleaved caspase-3 expression contrary to marked downregulation of p-PI3K, p-Akt, and p-GSK-3β, as compared with the normal group. Concomitantly, large multifocal areas of amyloid deposition associated with inflammatory tissue response were detected in the brains of ICV-STZ-injected mice. Normal animals which received MX-IV showed no significant difference in the formerly mentioned parameters, as compared with the normal group. ICV-STZ- injected animals which were treated with either the xanthone or donepezil showed obvious improvement in neuro-inflammation in addition to a marked reduction in amyloid plaques number and p-tau protein as well as cleaved caspase-3 expression owing to the prominent increase in p-PI3K, p-Akt, and p-GSK-3β expression exhibited by these groups, with MX-IV showing a comparable beneficial effect to the reference drug donepezil. Administration of ICV-STZ was stated to induce DNA damage that progresses to neuronal degeneration and death [41]. Animals receiving ICV-STZ injection, in the present investigation, have also shown an obvious decrease in neuronal survival rate in all brain areas. Treatment with either MX-IV or donepezil increased the neuronal survival rate, as compared with the ICV-STZ group, yet it was noticeably lower than that of the normal group in the cerebral cortex. In the cornu ammonis (CA) 4 and dentate gyrus (DG) regions of the hippocampus, MX-IV treatment showed a substantial decrease in the survival rate, as compared with the normal group; however, in the CA3 region, both MX-IV and donepezil-treated groups showed no significant difference in neuronal survival rate, as compared with normal mice. As for the histopathological examination, photomicrographs of normal animals, whether treated with MX-IV or not, showed no histopathological alterations. The ICV-STZ group showed diffuse gliosis with numerous scattered dark degenerated neurons in the cerebral cortex and the striatum. The cerebral cortical blood vessels also exhibited severe vasculitis and congestion associated with neuronal degeneration and neuronophagia with multifocal aggregations of lymphocytic infiltration. Several dark degenerated neurons were detected in the hippocampal regions CA3, CA4, and DG. Administration of MX-IV reversed STZ injection-induced deleterious effects, with the cerebral cortex showing apparently normal neurons, whereas the striatum showed diffuse gliosis in some sections. The hippocampus of this group revealed apparently normal neurons in all examined regions except for DG regions where few scattered dark degenerated neurons were detected. ICV-STZ-injected animals that received donepezil revealed apparently normal neurons of the cerebral cortex and the hippocampus, while the striatum showed diffuse gliosis in some instances.

### Limitations

The present study was concerned with investigating MX-IV neuroprotective effects in the ICV-STZ-induced AD model in adult male Swiss albino mice; yet further studies are required to empathize the favorable effects of MX-IV under different circumstances, such as AD transgenic animal models. Additionally, MX-IV-induced neuroprotection could be addressed in female mice as well as old ones to explore the impact of sex and age. Nonetheless, the current investigation selected the ICV-STZ model as a reputable method of AD induction which mimics the oxidative and neuroinflammatory pathological changes that precede the fully developed AD witnessed in old age which takes many years to become evident. Therefore, adult mice were deemed suitable for that study. Female gender was not included in that investigation as, actually, females are protected against AD via their high estrogen levels; thus, ovariectomy could be required to detect ICV-STZ associated neuropathological derangements in an obvious manner. Consequently, it is encouraged that other studies address these issues which could boost our study results regarding MX-IV neuroprotective potentials.

## 5. Conclusions

Treatment with MX-IV ameliorated ICV-STZ-provoked oxidative stress and neuro-inflammation-associated hazards, namely Aβ deposition, tau hyperphosphorylation, neuronal death, and cognitive impairment. This favorable neuro-protective effect can be attributed to MX-IV ability to regulate the PI3K/Akt/GSK-3β pathway which is a key player in AD pathogenesis and progression. Therefore, further investigations should be carried out to explore MX-IV as a possible candidate for AD management.

## Data Availability

Non-applicable.

## Supplementary Materials

The following are available online at https://www.mdpi.com/article/10.3390/biology10121298/s1, Table S1: NMR data of compounds mangostanaxanthone IV (MX-IV) (CDCl_3_, 850 and 214 MHz), Figure S1: ESIMS of MX-IV, Figure S2: ^1^H NMR spectrum of MX-IV in CDCl_3_ (850 MHz), Figure S3: ^13^C NMR spectrum of MX-IV in CDCl_3_ (214 MHz), Figure S4: HMBC spectrum of MX-IV, Figure S5: HSQC spectrum of MX-IV.
