# Supplementary material for: Mangostanaxanthone IV Ameliorates Streptozotocin-Induced Neuro-Inflammation, Amyloid Deposition, and Tau Hyperphosphorylation via Modulating PI3K/Akt/GSK-3β Pathway
Source: Biology (Basel). 2021 Dec 8;10(12):1298. doi: 10.3390/biology10121298 (PMC8698304; doi:10.3390/biology10121298)
Supplement: Supplementary file 1 [file biology-10-01298-s001.zip › biology-1452918-supplementary.pdf]

Mangostanaxanthone IV ameliorates streptozotocin-induced neuro-inflammation, amyloid deposition, and tau hyperphosphorylation via modulating PI3K/Akt/GSK-3β pathway

Hossam M. Abdallah [1,2,*], Nesrine S. El Sayed [3], Alaa Sirwi [1], Sabrin R. M. Ibrahim [4,5], Gamal A. Mohamed [1,6]
and Nora O. Abdel Rasheed [3]

[1] Department of Natural Products and Alternative Medicine, Faculty of Pharmacy, King Abdulaziz University, Jeddah 21589, Saudi Arabia; asirwi@kau.edu.sa (A.S.); gahussein@kau.edu.sa (G.A.M.)
[2] Department of Pharmacognosy, Faculty of Pharmacy, Cairo University, Giza 11562, Egypt
[3] Department of Pharmacology and Toxicology, Faculty of Pharmacy, Cairo University, Giza 11562, Egypt; nesrine.salah@pharma.cu.edu.eg (N.S.E.S.); nora.osama@pharma.cu.edu.eg (N.O.A.R.)
[4] Department of Chemistry, Preparatory Year Program, Batterjee Medical College, Jeddah 21442, Saudi Arabia; sabrin.ibrahim@bmc.edu.sa
[5] Department of Pharmacognosy, Faculty of Pharmacy, Assiut University, Assiut 71526, Egypt (sabreen.ibrahim@pharm.aun.edu.eg)
[6] Faculty of Pharmacy, Pharmacognosy Department, Al-Azhar University, Assiut Branch, Assiut 71524, Egypt.
*Correspondence: hmafifi@kau.edu.sa; Tel.: 00966544733110

Methodology:

Induction of SAD

To prevent cerebral vein penetration, the freehand ICV technique indicated by Pelleymounter and colleagues [1,2] and modified by Warnock [3] was used in the present study. The needle was introduced at the following co-ordinates from bregma; 1 mm mediolateral, -0.1 mm anteroposterior, and -3 mm dorsoventral. Mice started to exhibit normal behavior 1 min after the injection [4].

Morris water maze (MWM) test

The MWM test traces the animals' learning and visuospatial cognitive functions [5]. A large stainless steel circular pool (150 cm in diameter and 60 cm in height) was used in the present study. It was divided arbitrarily into four equal quadrants with the help of two perpendicular threads. A platform (10 cm width, 28 cm in height), painted in black, was constantly put inside the target quadrant of this pool. The pool was half filled with water which was made opaque using dye so that the platform becomes invisible. The procedure was carried out on five successive days [6]. Each mouse was subjected to two consecutive trials on the first 4 days of the

test, with an interval of at least 15 min between the trials. The maximum time for each trial was 120 s. If the animal managed to find the hidden platform within that time, it was kept there for extra 20 s before being removed. The mouse that failed to find the hidden platform during the designated time was gently guided onto the platform and kept there for 20 s. The mean escape latency (MEL) time which is the time taken by each mouse to find the hidden platform, was recorded for each mouse throughout each of the trials executed over the four testing days and was considered as an acquisition index [7]. On the test day, the time consumed by each mouse in the target quadrant where the hidden platform was previously positioned, was recorded as memory indicator [7,8].

Estimation of biochemical parameters
Mouse ELISA kits were used for the measurement of TNF-α (Cusabio, Wuhan, China) as well as GSH, MDA, H_2O_2, IL-6, and NADPH oxidase brain contents (My Bio Source, CA, USA). The assessment was performed according to the instructions supplied in each kit.

Characterization of isolated compound
Mangostanaxanthone IV**:** Yellow crystals, mp 219 ºC; $[\alpha]_D^{25}$ +14.1 (*c* 0.5, MeOH); UV (MeOH) λ_{max} (log ε) 248 (4.56), 318 (4.32), 356 (3.98) nm; IR (KBr disc) ν_{max} 3486, 2947, 1644, 1611, 1580, 1052 cm^{-1}; NMR spectral data, see Table 1; HR ESIMS *m/z* 427.1759 $[M+H]^+$ (calcd for $C_{24}H_{29}O_7$, 427.1757).

Table S1: NMR data of compounds **Mangostanaxanthone IV (**MX-IV**)** ($CDCl_3$, 850 and 214 MHz).

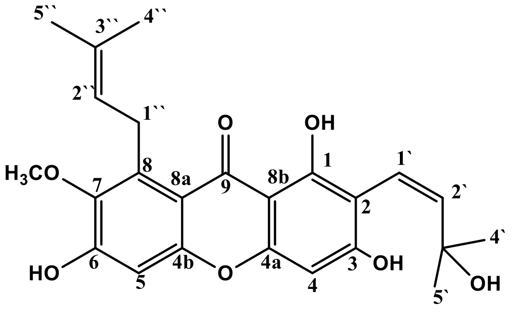

	MX-IV		
**No.**	**δH (mult., *J* (Hz)**	**δC (mult.)**	**HMBC**
1	-	157.9 s	-
2	-	104.5 s	-
3	-	159.9 s	-
4	6.20 s	94.2 d	2, 3, 4a, 8b
4a	-	156.3 s	-
4b	-	155.7 s	-
5	6.82 s	101.7 d	6, 7, 4b, 8a
6	-	154.7 s	-
7	-	142.7 s	-
8	-	137.0 s	-
8a	-	112.2 s	-
8b	-	103.7 s	-
9	-	182.0 s	-
1-OH	13.70 s	-	1, 8b
3-OCH_3	-	-	-
7-OCH_3	3.79 s	62.0 q	7
1`	6.72 d (9.6)	115.7 d	2, 3, 2`, 3`, 4`, 5`
2`	5.57 d (9.6)	127.2 d	1, 2, 3`, 4`, 5`
3`	-	77.9 C	-
4`	1.46 s	28.3 q	2`, 3`, 5`
5`	1.46 s	28.3 q	2`, 3`, 4`
1``	4.08 d (7.8)	26.6 t	7, 8, 8a, 2``, 3``
2``	5.25 tq (7.8, 1.8)	123.1 d	8, 4``, 5``
3``	-	132.0 s	-
4``	1.68 s	25.8 q	2``, 3``
5``	1.82 s	18.2 q	2``, 3``

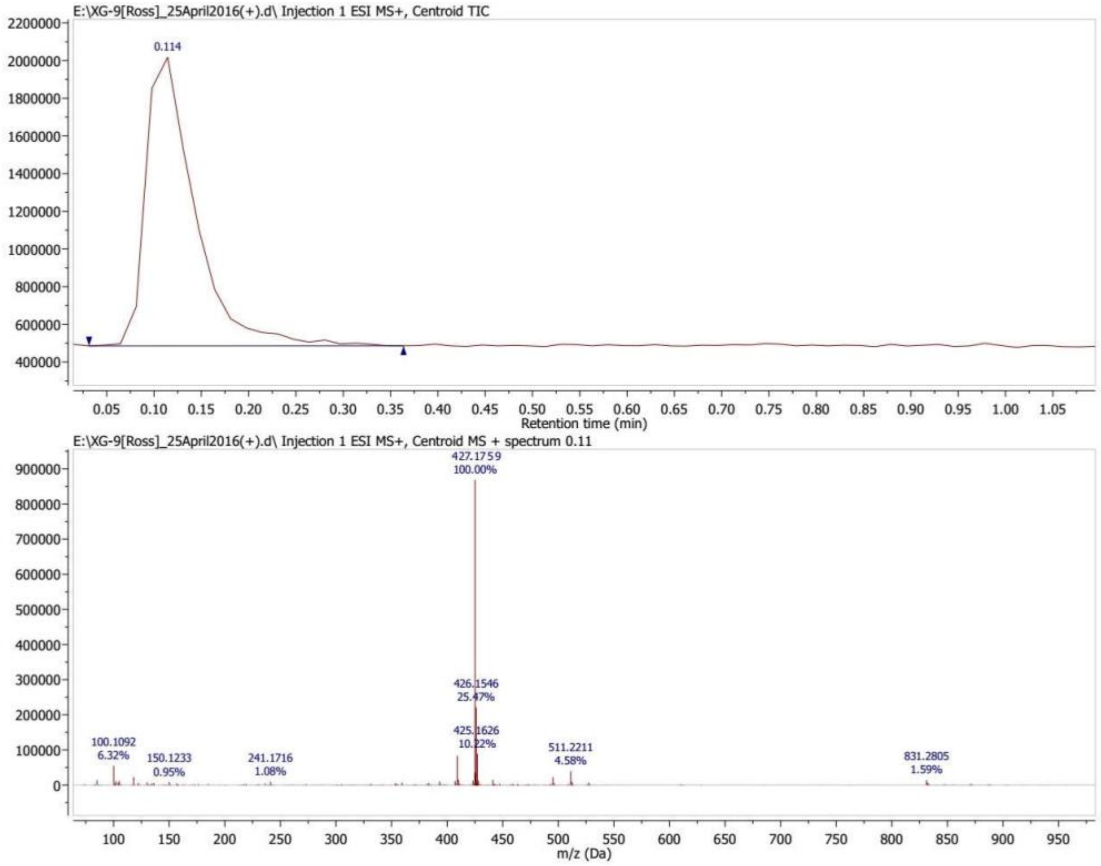

Figure S1: High resolution mass spectra of compound MX-IV

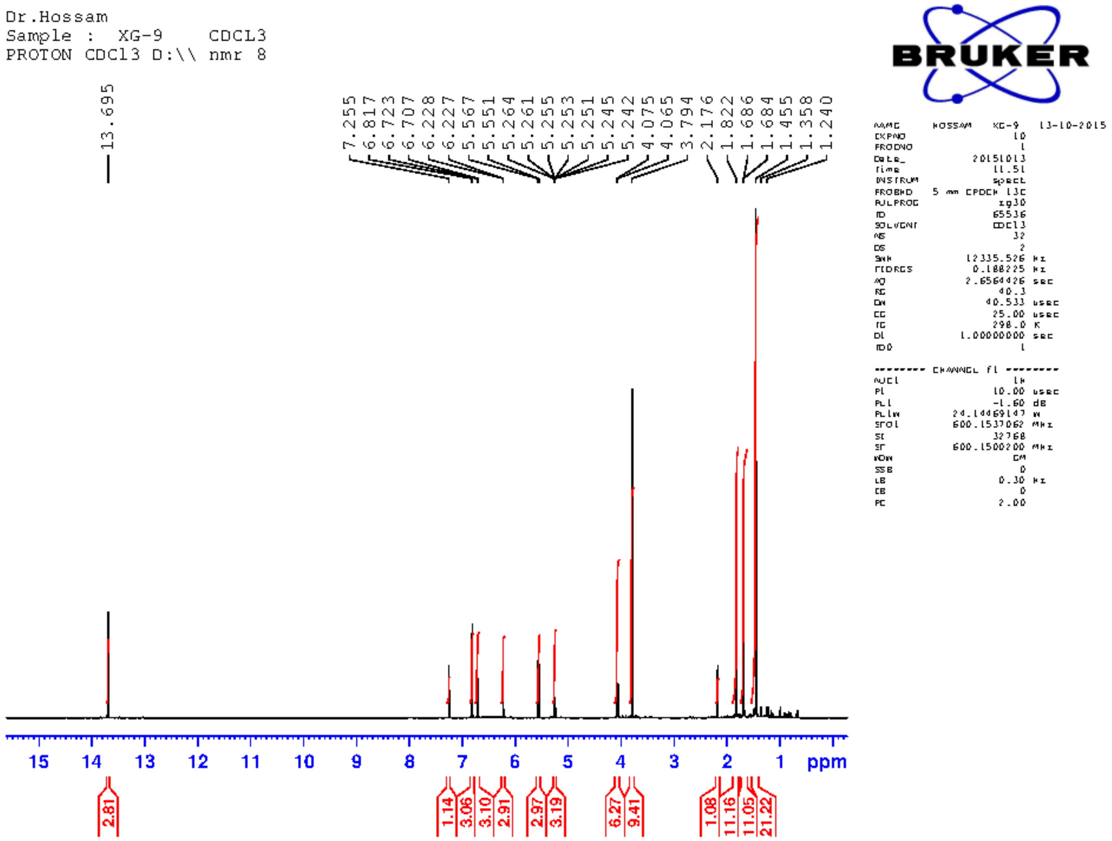

Figure S2: ^{1}H NMR spectra of compound MX-IV in $CDCl_3$

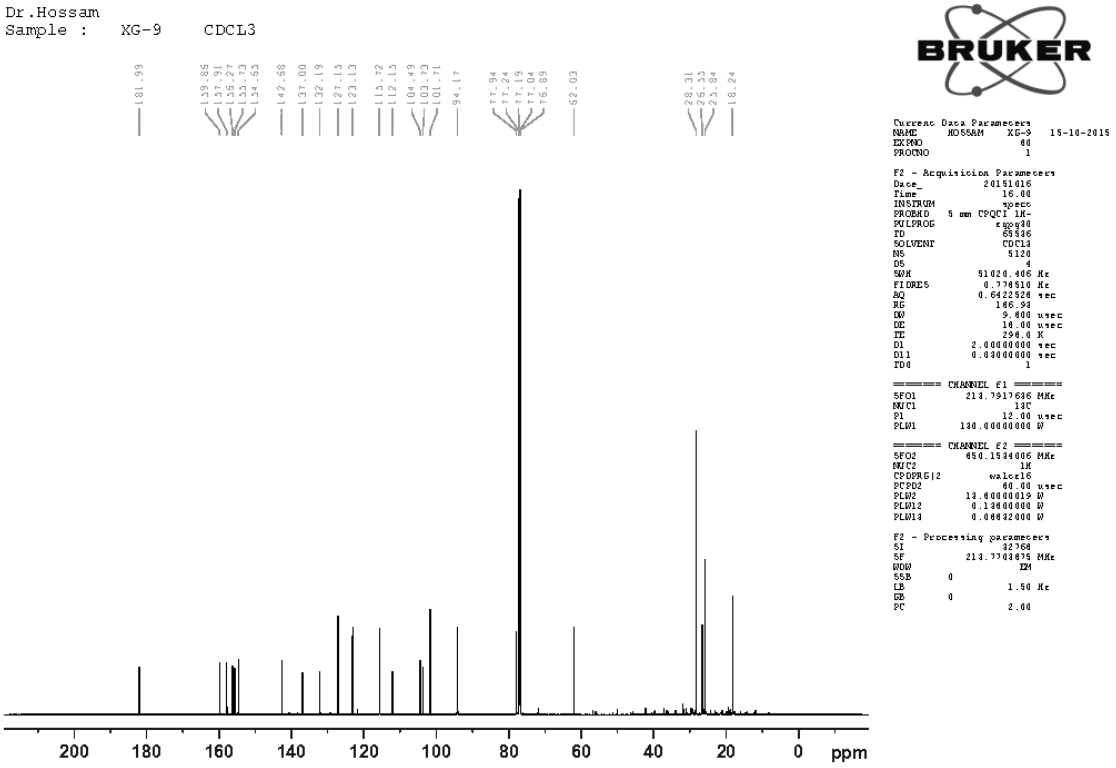

Figure S3: ^{13}C NMR spectra of compound MX-IV in $CDCl_3$

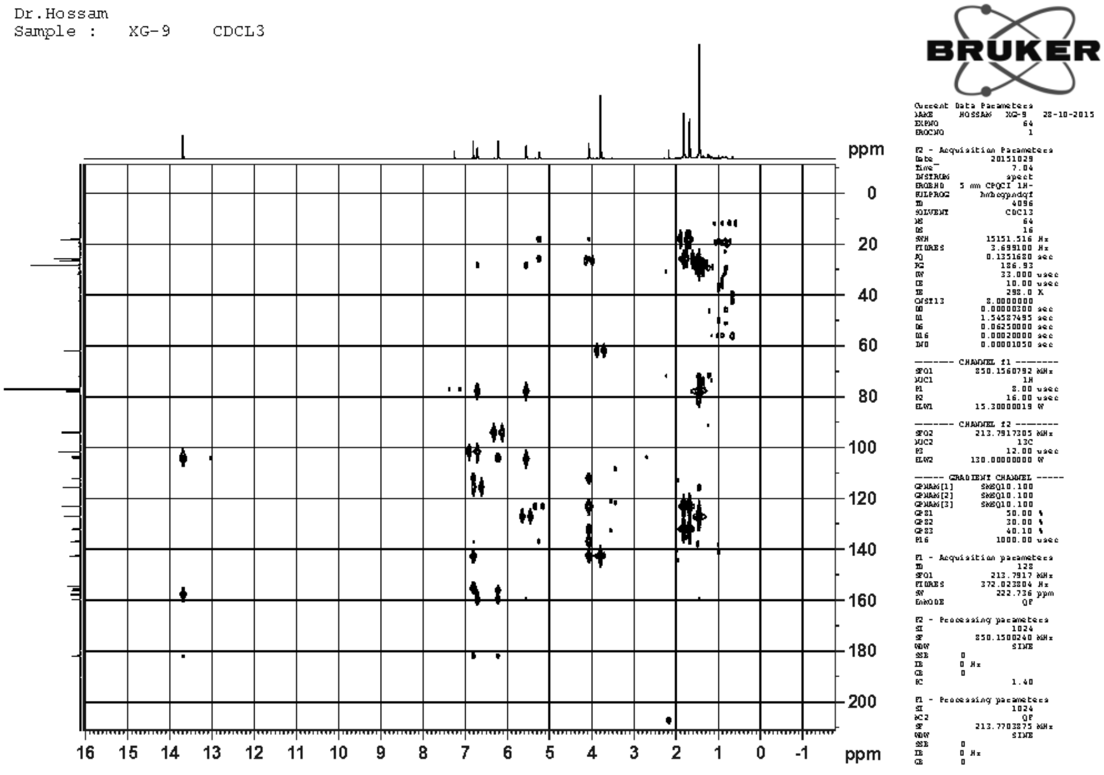

Figure S4: HMBC spectra of compound MX-IV in $CDCl_3$

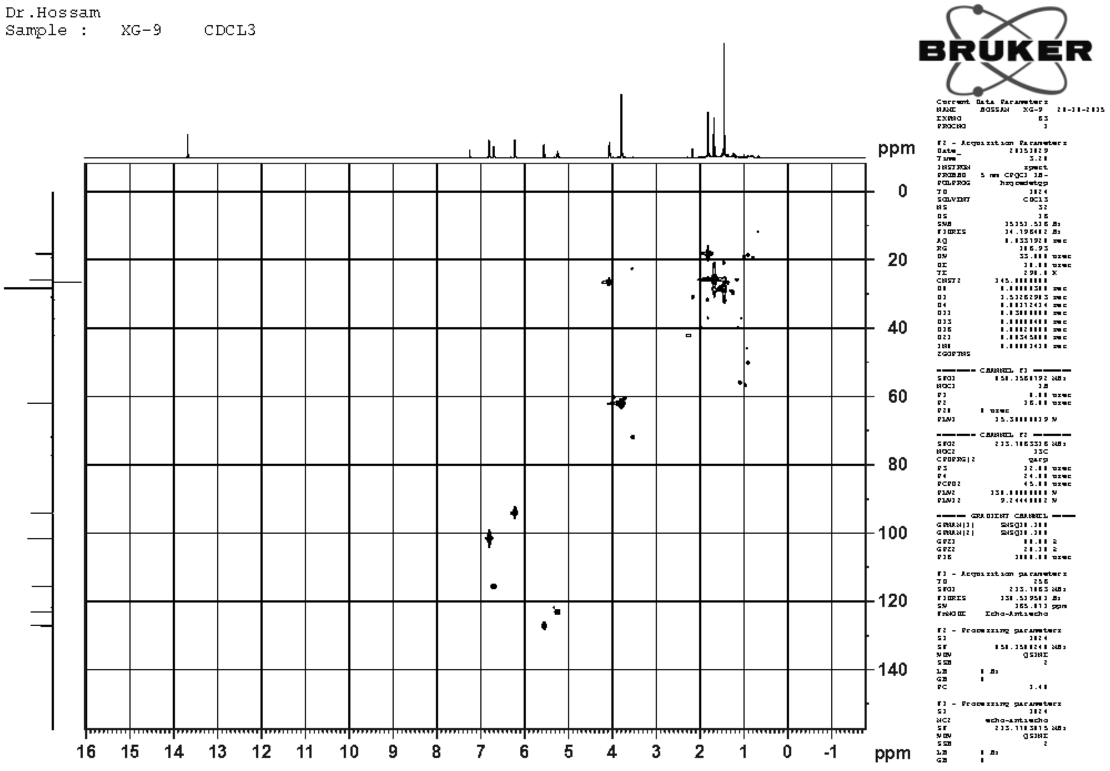

Figure S5: HSQC spectra of compound MX-IV in $CDCl_3$

References

1. Pelleymounter, M.A.; Joppa, M.; Carmouche, M.; Cullen, M.J.; Brown, B.; Murphy, B.; Grigoriadis, D.E.; Ling, N.; Foster, A.C. Role of corticotropin-releasing factor (CRF) receptors in the anorexic syndrome induced by CRF. *J. Pharmacol. Exp. Ther.* **2000**, *293*, 799-806.
2. Pelleymounter, M.A.; Joppa, M.; Ling, N.; Foster, A.C. Pharmacological evidence supporting a role for central corticotropin-releasing factor2 receptors in behavioral, but not endocrine, response to environmental stress. *J. Pharmacol. Exp. Ther.* **2002**, *302*, 145-152.
3. Warnock, G.I. Study of the central corticotrophin-releasing factor system using the 2-deoxyglucose method for measurement of local cerebral glucose utilisation. University of Bath Bath, 2007.
4. Cunha, M.P.; Pazini, F.L.; Rosa, J.M.; Ramos-Hryb, A.B.; Oliveira, Á.; Kaster, M.P.; Rodrigues, A.L.S. Creatine, similarly to ketamine, affords antidepressant-like effects in the tail suspension test via adenosine A 1 and A 2A receptor activation. *Purinergic Signal.* **2015**, *11*, 215-227.
5. D'Hooge, R.; De Deyn, P.P. Applications of the Morris water maze in the study of learning and memory. *Brain Res. Rev.* **2001**, *36*, 60-90.
6. Gupta, R.; Gupta, L.K. Improvement in long term and visuo-spatial memory following chronic pioglitazone in mouse model of Alzheimer's disease. *Pharmacol. Biochem. Behav.* **2012**, *102*, 184-190.
7. Singh, B.; Sharma, B.; Jaggi, A.S.; Singh, N. Attenuating effect of lisinopril and telmisartan in intracerebroventricular streptozotocin induced experimental dementia of Alzheimer's disease type: possible involvement of PPAR-γ agonistic property. *J. Renin Angiotensin Aldosterone Syst.* **2013**, *14*, 124-136.
8. Blokland, A.; Geraerts, E.; Been, M. A detailed analysis of rats' spatial memory in a probe trial of a Morris task. *Behavioural brain research* **2004**, *154*, 71-75.
